# Revisiting remote drivers of the 2014 drought in South-Eastern Brazil

**DOI:** 10.1007/s00382-020-05442-9

**Published:** 2020-09-03

**Authors:** Kathrin Finke, Bernat Jiménez-Esteve, Andréa S. Taschetto, Caroline C. Ummenhofer, Karl Bumke, Daniela I. V. Domeisen

**Affiliations:** 1 Department of Meteorology, Stockholm University, Stockholm, Sweden; 2 Institute for Atmospheric and Climate Science, ETH Zurich, Zurich, Switzerland; 3 Climate Change Research Centre, University of New South Wales, Sydney, Australia; 4 ARC Centre of Excellence for Climate Extremes, University of New South Wales, Sydney, Australia; 5 Department of Physical Oceanography, Woods Hole Oceanographic Institution, Woods Hole, USA; 6 Marine Meteorology Department, GEOMAR Helmholtz Centre for Ocean Research Kiel, Kiel, Germany

**Keywords:** Brazil 2014 drought, Teleconnection, ENSO, Blocking, MJO

## Abstract

South-Eastern Brazil experienced a devastating drought associated with significant agricultural losses in austral summer 2014. The drought was linked to the development of a quasi-stationary anticyclone in the South Atlantic in early 2014 that affected local precipitation patterns over South-East Brazil. Previous studies have suggested that the unusual blocking was triggered by tropical Pacific sea surface temperature (SST) anomalies and, more recently, by convection over the Indian Ocean related to the Madden–Julian Oscillation. Further investigation of the proposed teleconnections appears crucial for anticipating future economic impacts. In this study, we use numerical experiments with an idealized atmospheric general circulation model forced with the observed 2013/2014 SST anomalies in different ocean basins to understand the dominant mechanism that initiated the 2014 South Atlantic anticyclonic anomaly. We show that a forcing with global 2013/2014 SST anomalies enhances the chance for the occurrence of positive geopotential height anomalies in the South Atlantic. However, further sensitivity experiments with SST forcings in separate ocean basins suggest that neither the Indian Ocean nor tropical Pacific SST anomalies alone have contributed significantly to the anomalous atmospheric circulation that led to the 2014 South-East Brazil drought. The model study rather points to an important role of remote forcing from the South Pacific, local South Atlantic SSTs, and internal atmospheric variability in driving the persistent blocking over the South Atlantic.

**Electronic supplementary material:**

The online version of this article (10.1007/s00382-020-05442-9) contains supplementary material, which is available to authorized users.

## Introduction

Agriculture represents an important part of Brazil’s economy. Products differ regionally depending on the local climate. The climate in South-Eastern (SE) Brazil, particularly in the states Minas Gerais and Espírito Santo, provides ideal growing conditions favorable for the development of large coffee plantations (de Camargo 2010). Brazil evolved into the largest coffee exporter in the world (Varangis et al. 2003), with the country’s economy dependent on the crop’s productive yield. However, coffee plants are very sensitive to weather, especially to fluctuations of temperature and precipitation (de Camargo 2010). In January and February (JF) 2014, insufficient precipitation, strongly lacking soil moisture and unusually high temperatures (Blunden and Arndt 2015; Nobre et al. 2016; Silva et al. 2015) led to a decline in coffee harvest by 15–40% (Nobre et al. 2016). Reduced rainfall, frequently recorded during the past decades (Coelho et al. 2016), as well as past and projected warming temperatures with climate change (Intergovernmental Panel on Climate Change 2013; Koh et al. 2020) make the region increasingly vulnerable to drought conditions. Therefore, understanding potential local and remote relationships between different atmospheric circulation patterns that have the ability to substantially affect the agricultural sector of Brazil (e.g. Anderson et al. 2019) is extremely important.

Austral summer is the peak of the wet season and precipitation in SE Brazil is dominantly influenced by the variability of the South Atlantic Convergence Zone (SACZ) (Kodama 1992), which is a northwest–southeast oriented convection band stretching from the Amazon Basin towards SE Brazil (Nogués-Paegle and Mo 1997; Carvalho et al. 2004). Its characteristic convective activity significantly contributes to the precipitation amount during the South American monsoon season (Kodama 1992). Together with SE South America, SE Brazil forms a regional dipole characterized by spatially alternating enhanced and reduced precipitation, whose variability is strong on intraseasonal time scales (e.g. Paegle et al. 2000; Carvalho et al. 2002; Boers et al. 2014). The configuration and strength of the South American Low-Level Jet (SALLJ), which usually provides moisture and precipitation from the Amazon Basin to regions further south, can strongly modulate the SACZ’s moisture supply (Nogués-Paegle and Mo 1997; Liebmann et al. 2004). Liebmann et al. (2004), for example, find an unusually strong SALLJ linked to enhanced precipitation in southern Brazil, while SE Brazil is unusually dry due to an anticyclonic anomaly forming off the east coast of SE South America. Their study emphasizes the importance of the phase with which a Rossby wave train, originating in the mid-latitude Pacific, approaches the Andes in generating SACZ rainfall anomalies (e.g. Liebmann et al. 2004).

From a remote perspective, processes operating on various time-scales can influence the South American monsoon system (Marengo et al. 2012, and references therein). The intra-seasonal variability of the SACZ can be strongly modulated by wave trains excited by the Madden–Julian Oscillation (MJO—Madden and Julian 1971, 1972) (e.g. Carvalho et al. 2004). The eastward progression of the MJO’s characteristic convective dipole over the western Indian Ocean toward the maritime continent has been associated with a switch from wet to dry conditions in SE Brazil, accompanied by an anticyclonic circulation over the South Atlantic during austral summer (e.g. Vera et al. 2018). MJO-related convection over the maritime continent is found to roughly coincide with reduced SACZ precipitation (Carvalho et al. 2004; Liebmann et al. 2004; Alvarez et al. 2016; Barreiro et al. 2019). Rodrigues and Woollings (2017) link eastward propagating Rossby wave packets initiated by convection over the Indian Ocean with negative precipitation anomalies related to South Atlantic blocking and suppressed SACZ episodes with a time lag of about 6 days.

On inter-annual timescales, the El Niño-Southern Oscillation (ENSO) is the main large-scale mode of variability affecting South American climate (Ropelewski and Halpert 1987; Cai et al. 2020). In austral summer 2013/2014, the bimonthly Multivariate ENSO Index (MEI from the National Oceanographic and Atmospheric Administration; Wolter and Timlin 1993, 1998) indicated neutral conditions in the tropical Pacific, while the Niño3.4 index (Trenberth 1997) suggested weak La Niña SST characteristics with values slightly below the threshold of $-0.4\,^\circ \text{C}$ for JF. While the affected region is not clearly associated with a dry or wet tendency during El Niño and La Niña events, Grimm (2004) and Tedeschi et al. (2015) suggest that the region is prone to dryness during the summer of La Niña periods, especially during January. Moisture flux divergence accompanied by low-level anticyclonic anomalies emerging from excess precipitation during austral spring and the corresponding anomalous surface cooling in the SACZ region are then suggested to limit precipitation in SE Brazil during January (Grimm 2004; Tedeschi et al. 2015).

In JF of 2014, SE Brazil experienced severe drought conditions. An anomalous blocking anticyclone extended the South Atlantic subtropical high’s western boundary over land (Seth et al. 2015). This anomalous mid-tropospheric anticyclone persisted for a remarkable 5–6 weeks and influenced the atmospheric circulation by blocking the passage of cyclones usually migrating from the south to the area of interest (Seth et al. 2015; Silva et al. 2015; Coelho et al. 2016; Nobre et al. 2016). Additionally, moist air from the Amazon Basin, usually transported towards SE Brazil by the SALLJ, was redirected, leading to high positive precipitation anomalies east of the Andes and farther south of the study area (Silva et al. 2015; Nobre et al. 2016). Inhibited convective activity due to descending air over SE Brazil led to the absence of SACZ episodes in early 2014 (Silva et al. 2015; Coelho et al. 2016; Nobre et al. 2016), making the drought more severe (Nobre et al. 2016). Consistent with the above described influence of the anticyclonic anomaly on the South American circulation, SE Brazil precipitation has been found to negatively correlate with the number of blocking days in subtropical South America (Rodrigues and Woollings 2017). Persisting warm South Atlantic SSTs evolved into a marine heatwave during the 2014 blocking event as a result of the anticyclone in the South Atlantic via increased solar radiation on the ocean’s surface due to low cloudiness (Rodrigues et al. 2019). However, it is unclear if the marine heatwave event played a role in the persistence of the 2014 South Atlantic height anomaly. In fact, Zou et al. (2018) and Barreiro et al. (2019) suggest that the warm SST conditions in the South Atlantic positively fed back onto the existing surface conditions in SE Brazil.

Western tropical Pacific SSTs (Seth et al. 2015) and concurrent unusual convective heating (Seth et al. 2015; Coelho et al. 2016) have been suggested as a possible source for an induced atmospheric teleconnection across the South Pacific that influenced South American hydroclimatic conditions in 2014. Coelho et al. (2016) argued that an interaction between the tropical and extratropical Pacific influenced the circulation anomalies that propagated to South America. More specifically, Walker circulation anomalies developed with ascending motion north-east of Australia and descending motion in the central-eastern tropical Pacific. These vertical motions interacted with the meridional Hadley cell, producing a stationary atmospheric Rossby wave that reinforced unusually high pressure throughout the lower and middle troposphere off the east and west coasts of South America (Coelho et al. 2016). Seth et al. (2015) relate a geopotential height pattern as observed in 2014 over the South Atlantic and SE South America with convection triggered by anomalous warming in the western tropical Pacific and propose a relation to the negative phase of the Inter-decadal Pacific Oscillation. More recently, deep convection over the eastern Indian Ocean associated with MJO events has been linked with blocking episodes over the South Atlantic (Rodrigues and Woollings 2017), such as those observed in 2014 (Rodrigues et al. 2019) and 2017 (Manta et al. 2018). Hence, various potential origins of the signal that initiated the anticyclonic anomaly over the South Atlantic have been suggested by previous studies. In this study, we use a combination of observational analyses and idealized sensitivity experiments to further investigate the proposed origins of the South Atlantic anticyclonic pattern that caused the 2014 drought in SE Brazil.

## Data and methods

### Study area

Geographically, the study area covers most of the SE region of Brazil, including the states of Minas Gerais, Espírito Santo, Rio de Janeiro and part of São Paulo. It is bounded by the grid points closest to 40–$50^\circ \text{W}$ and 15–$23^\circ \text{S}$ and it is indicated by a framed box over South America throughout the figures in this study. This particular region exhibited a pronounced rainfall deficit in JF 2014. For analyzing the JF 500 hPa geopotential height (500GPH) anomalies that were part of the anticyclonic blocking pattern in 2014, the area $25^\circ \text{S}$–$37.5^\circ \text{S}$ and $25^\circ \text{W}$–$50^\circ \text{W}$ was chosen.

### Reanalysis and observational data

For the synoptic overview of the 2014 drought in SE Brazil precipitation, 500GPH, sea level pressure (SLP), and SST were analyzed. The enhanced version of the CMAP global gridded dataset, provided by the Climate Prediction Center (CPC) with a horizontal resolution of $2.5^\circ \times$ $2.5^\circ$ (Xie and Arkin 1997) was used for precipitation. The monthly precipitation amounts are given in mm/day and are available over a 41-year period from 1979 to 2019. Monthly SST anomalies, computed on a $2^\circ$ latitude-longitude grid, are based on the National Oceanic Administration’s (NOAA) Extended Reconstructed Sea Surface Temperature (ERSST) v4 data from 1958 to 2019 (Huang et al. 2015). Daily SST anomalies are based on NOAA High-resolution Blended Analysis for Sea surface temperature (Reynolds et al. 2007) with a $0.25^\circ$ latitude-longitude grid that is available from 1982 to 2019. SLP and 500GPH fields are derived from the ERA-Interim reanalysis dataset provided by the European Centre for Medium-Range Weather Forecasts (Dee et al. 2011). Global data have been retrieved for the chosen 41-year time period from 1979 to 2019 with a horizontal resolution of $2.5^\circ \times$ $2.5^\circ$ for both monthly and daily data. Daily mean outgoing longwave radiation (OLR) data, given on a $2.5^\circ \times$ $2.5^\circ$ horizontal resolution and based on the NOAA Interpolated Outgoing Longwave Radiation data set (Liebmann and Smith 1996) are retrieved for the time period 1979–2019. The monthly (daily) anomalies were calculated as the deviation from the long-term monthly (daily) means. Anomalies above the 95th percentile and below the 5th percentile are considered significant. All analyses use the longest available time period available for the respective data set given above. The Niño3.4 index based on the HadISST dataset was used to identify ENSO SST characteristics in the tropical Pacific, while the Real-time Multivariate MJO (RMM) index (Wheeler and Hendon 2004) is used to characterize the state of the MJO.

### Statistical analysis

For the correlation analysis, the datasets were detrended using a linear trend for the available 41-year time period. Additionally, the climatological values of JF were subtracted from each year’s monthly mean, respectively, before averaging over the 2 months and the respective study area to create an index. The Pearson correlation coefficient was then used to examine the relationship between the South Atlantic 500GPH and SE Brazil precipitation anomalies as well as between South Atlantic 500GPH, South Atlantic SSTs and SE Brazil OLR anomalies. Additionally, statistically significant composite precipitation and 500GPH anomalies in Fig. 3 are determined by using the 95th and 5th percentile based on 1000 bootstrap samples and are marked with dots.

### Model simulations

The role of SSTs in various locations during the 2014 Brazilian drought is examined using Isca (Vallis et al. 2018). Isca is an atmospheric modelling framework that allows for the idealized modelling of the global circulation. It has been used to simulate a wide range of atmospheric processes, including SST forced atmospheric teleconnections (e.g.  Thomson and Vallis 2018; Jiménez-Esteve and Domeisen 2019). It uses the Geophysical Fluid Dynamics Laboratory (GFDL) dynamical core. Here, we use the same general model configuration as in Jiménez-Esteve and Domeisen (2019). In this configuration, simple moist processes are considered through evaporation from the surface and fast condensation (i.e. no explicit liquid water content in the atmosphere) which interacts with the radiation and the convection scheme. We use realistic topography and the continental outline from the ECMWF model (Dee et al. 2011). The land-sea contrast is achieved by changing characteristics such as the mixed layer depth, evaporation resistance and albedo as in Thomson and Vallis (2018). We use a Gaussian grid with a T42 spectral resolution (corresponding to a $2.79^\circ$ horizontal resolution) and 50 vertical levels up to 0.02 hPa.

The experiments consist of a climatological run where SSTs are prescribed following the 1958–2016 climatological monthly mean seasonal cycle SST from NOAA ERSSTv4 (Huang et al. 2015), and five sensitivity experiments where SSTs follow the 2013–2014 austral summer evolution, i.e. 2013 anomalies are imposed from August 2013 to April 2014, while May to July 2014 SSTs are set to climatology. Monthly SST anomalies are linearly interpolated to daily values in the model to obtain a smooth cycle. Note that this might exclude SST variations on shorter time scales. In the first sensitivity experiment SSTs are forced in all ocean basins. In the other four sensitivity experiments, 2013/2014 SST anomalies are imposed in the Indian Ocean [Box A: $30^\circ \text{N}$–$50^\circ \text{S}$ and $45^\circ \text{E}$–$110^\circ \text{E}$], the tropical Pacific [Box B: $15^\circ \text{N}$–$20^\circ \text{S}$ and $125^\circ \text{E}$–$285^\circ \text{E}$], the extratropical South Pacific [Box C: $20^\circ \text{S}$–$65^\circ \text{S}$ and $125^\circ \text{E}$–$285^\circ \text{E}$], and the South Atlantic [Box D: $10^\circ \text{S}$–$70^\circ \text{S}$ and $285^\circ \text{E}$–$360^\circ \text{E}$], respectively (the areas are marked in Fig. 2d). We choose these domains in order to capture the main SST anomalies that could have influenced the atmospheric conditions during JF 2014 based on previous findings. The atmospheric 500 hPa geopotential height response for a given SST anomaly experiment is computed as the difference between the climatological run and the respective 2013/2014 SST anomaly run. The climatological simulation is integrated for 150 years with a repeating SST seasonal cycle forcing, while the atmosphere is allowed to evolve freely, creating an ensemble of model simulations. The first 20 years are removed as spin-up. The five 2013–2014 anomaly SST simulations are initialized from the 20th year of the climatological simulation and are forced with the 2013/2014 SST field for another 40 years, removing the first year as spin-up. This yields 39 ensemble members for each of the sensitivity experiments.

## Results

### Observed conditions in January–February 2014

**Fig. 1:**
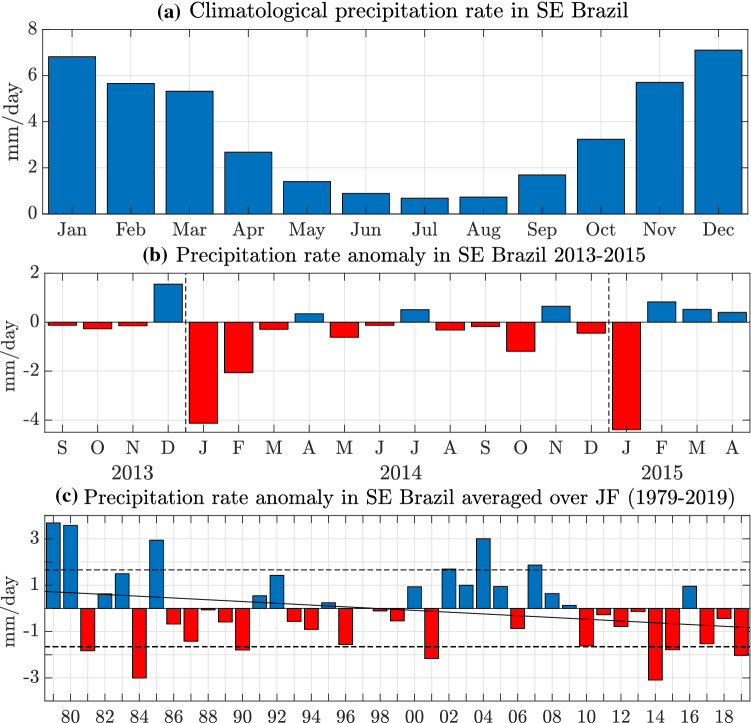
**a** Climatological precipitation rate averaged over the study area in SE Brazil. **b** Precipitation rate anomalies averaged over the study area in SE Brazil from September 2013 to April 2015 relative to the seasonal cycle for all years. **c** Seasonally averaged precipitation rate anomalies during JF within the study area in SE Brazil for the time period from 1979 to 2019. The dashed black lines indicate the corresponding standard deviation of ± 1.65 mm/day. The solid line displays the linear trend. Values are based on the enhanced CMAP dataset

**Fig. 2:**
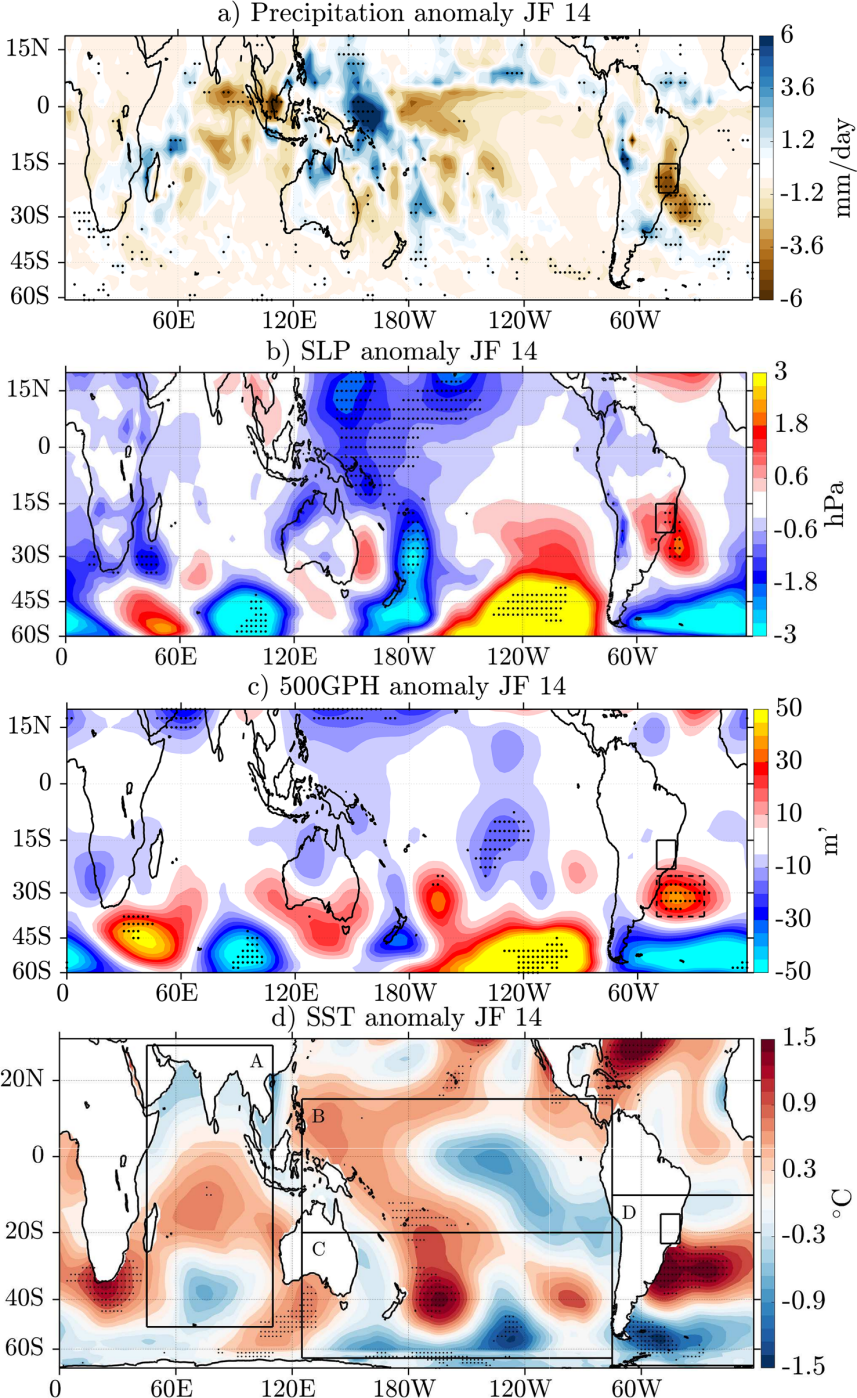
Two-monthly composite anomalies for JF 2014 for **a** precipitation rate (in mm/day), **b** SLP (in hPa), **c** 500GPH (in m$^{\prime }$), **d** SST (in $^\circ \text{C}$). The data in **a** is based on CMAP, **b** and **c** are based on the ERA-Interim reanalysis and **d** is derived from ERSSTv4. SLP, 500GPH and precipitation datasets are analyzed for the time period from 1979 to 2019 while data from 1958 to 2019 are used for SSTs. The dotted anomalies reflect significance at the 5% level. The black box over South America indicates the study area in SE Brazil. The dashed black box over the South Atlantic in **c** indicates the area associated with the blocking anticyclone. Boxes A–D in **d** indicate the areas of the 2013/2014 SST anomalies in the Indian Ocean, the tropical and South Pacific as well as the Atlantic that are used as forcing in the model experiments

SE Brazil has two distinctive seasons, clearly recognisable in Fig. 1a, which shows the climatological precipitation rates spatially averaged over the study area (see Fig. 2, black box over SE Brazil). The wet season lasts from October to March with precipitation rates between 5 and 7 mm/day. During the dry period from April to September, the precipitation rate decreases to about 0.7 mm/day at its lowest point. Figure 1b exhibits the corresponding precipitation anomalies in SE Brazil from September 2013 to April 2015. The months leading into the 2014 drought do not show exceptionally dry values, with the preceding December being an unusually wet month instead. In JF 2014 the anomalies switch sign, yielding extremely low precipitation in the study area. Unusual dryness persists for most of the year with an exceptionally deficient start to the wet season in 2014/2015. The remarkable precipitation shortage together with unusually warm temperatures and the consequently emerging high water demand in one of the most populated regions in South America in early 2014 were mainly responsible for the aggravated drought conditions in SE Brazil (Nobre et al. 2016); this period is studied in greater detail. Figure 1c shows JF precipitation anomalies, averaged over the study area in SE Brazil from 1979 to 2019. Seven of the 41 years are considered unusually dry and surpass the standard deviation of $-1.65$ mm/day. The year 2014 is the driest year over the full record with 3.1 mm/day below average, i.e. a rainfall deficit of almost 2 standard deviations below the long-term mean. Note that none of the seven driest summers is followed by another exceptionally dry event during the following summer, except for 2014.

For the analysis of the atmospheric conditions in JF 2014 in SE Brazil, the anomalous distributions of precipitation, SLP, 500GPH and SST are evaluated (Fig. 2). In JF 2014, the precipitation for the entire study area, which is marked as the black box over SE Brazil, is below average (Fig. 2a). The south-western part of the study area was most strongly affected with considerable rainfall deficits in excess of $-5$ mm/day. East of the Andes, there is a substantial wet anomaly with values above 9 mm/day. Uruguay and central Argentina also experienced anomalously wet conditions (Fig. 2a). Furthermore, significant convection is found over the western tropical Pacific during JF 2014, potentially generating a source for Rossby waves to leave the tropics. The anomalous precipitation in SE Brazil is accompanied by substantial positive SLP anomalies (Fig. 2b) with values up to 2 hPa between 15 and $30^\circ \text{S}$ over SE Brazil and the South Atlantic. Another strong positive SLP anomaly is located in the South Pacific Ocean. The distribution of 500GPH anomalies (Fig. 2c) exhibits similarities to the SLP anomaly pattern. The positive anomalies in the Atlantic at approximately $60^\circ \text{W}$–$10^\circ \text{W}$ centered at about $30^\circ \text{S}$ are visible in both variables, indicating a barotropic structure in the lower troposphere that blocked the atmospheric circulation at all heights below 500 hPa. Hence, cyclones and their corresponding fronts that usually form in the southern mid-latitudes and move northward towards the study area were redirected and could not provide precipitation to SE Brazil. Additionally, the wet anomaly east of the Andes indicates that moist air, produced in the Amazon Basin, did not reach the study area and therefore could not contribute to the precipitation. The fact that the strong height and precipitation anomalies are visible in these two-monthly averages emphasizes the persistence of this unusual event in 2014. The SST anomalies are displayed in Fig. 2d. Unusually warm SST anomalies developed a severe marine heatwave in the south-western Atlantic Ocean (Rodrigues et al. 2019), co-located with the anticyclone. In most of the western tropical Pacific positive anomalies are found as well. In the eastern tropical Pacific, SSTs are colder than the climatological mean. This observed pattern in the tropical Pacific during JF 2014 resembles La Niña SST characteristics reflecting the Niño3.4 index slightly exceeding the threshold of $-0.4\,^\circ \text{C}$ during JF 2014.

### The role of the South Atlantic anticyclone

Grimm (2004) relates drier conditions in SE Brazil accompanied by low-level anticyclonic circulation over SE Brazil during January to a lagged response emerging from the tropical Pacific during the preceding spring of La Niña events. In JF 2014, a weak La Niña-like SST pattern was observed in the tropical Pacific. These tropical Pacific SST anomalies, accompanied by enhanced convection in the western tropical Pacific, are proposed to have contributed to the circulation anomalies that caused the drought in 2014 (e.g. Coelho et al. 2016). Apart from tropical Pacific SSTs, Rodrigues and Woollings (2017) identified convection over the eastern Indian Ocean, associated with the MJO, to trigger Rossby wave trains potentially leading to blocking in the South Atlantic. Interestingly, according to the RMM index, the MJO phases conducive to atmospheric blocking in SE Brazil were only briefly active in the beginning of the December to February season, but neither during the blocking nor in the two weeks preceding the event in 2014 (see Figure S1 in the Supplementary Material for the MJO phase evolution from December 2013 to February 2014). This suggests that although the MJO could have initiated the South Atlantic blocking event, external mechanisms may have contributed to the persistence of the blocking. Remote influences on the 500GPH over the South Atlantic from the tropical Pacific SSTs are thus further investigated in this study.

**Fig. 3:**
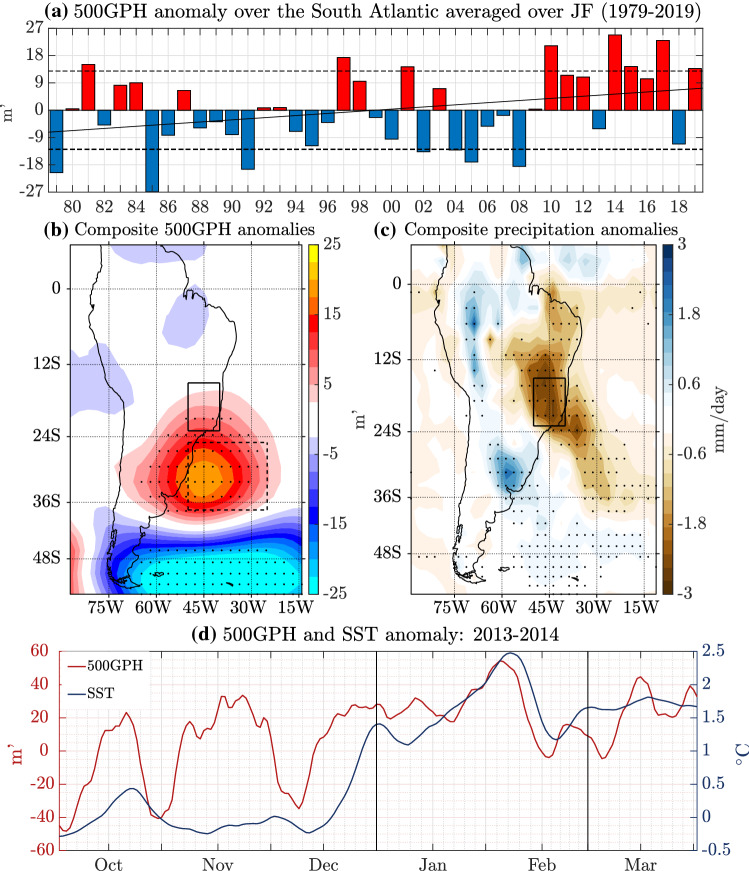
**a** 500GPH anomalies within the dashed box over the South Atlantic for JF 1979–2019. Data are based on the ERA-Interim reanalysis and are given in m$^{\prime }$. The dashed black lines display the corresponding standard deviation of $\pm 12.29$ m$^{\prime }$. The solid line reflects the linear trend. **b** Composite JF 500GPH anomalies for years with SE Brazil precipitation below one standard deviation (1981, 1984, 1990, 2001, 2014, 2015, 2019). **c** Composite JF precipitation anomalies for years with South Atlantic 500GPH above one standard deviation (1981, 1997, 2001, 2010, 2014, 2015, 2017, 2019). Dotted anomalies reflect significance at the 5% level based on 1000 bootstrap samples. **d** Daily evolution of South Atlantic 500GPH [$m^\prime$] (red; left axis) and SST [$^\circ C$] (blue; right axis) anomalies from October 2013 to March 2014. A 10-day running mean has been applied to the data. Vertical black lines encompass the JF time period

As a first step, the relation between the South Atlantic 500GPH and SE Brazil precipitation is examined. The South Atlantic 500GPH anomalies for JF (dashed box in Fig. 2c) for 1979–2019 are found to surpass the standard deviation of $\pm 12.29$ m$^{\prime }$ for eight out of 41 years (Fig. 3a). Seven out of these eight years agree with years with anomalously low precipitation in Fig. 1c while 6 out of the 7 years with anomalously low precipitation agree with anomalously enhanced 500GPH. With a positive anomaly of 23.27 m$^{\prime }$, 2014 exhibits the highest 500GPH anomaly over the South Atlantic over the full time period considered, almost exceeding 2 standard deviations. The Pearson correlation coefficient between the detrended time series of the South Atlantic 500GPH (Fig. 3a) and the SE Brazil precipitation anomalies (Fig. 1c) for the time period 1979 to 2019 is statistically significant with a value of $-0.55$ ($\text{p}=1.99 \times 10^{-4}$). Thus, deficient precipitation tends to coincide with high 500GPH and vice versa, explaining about 30% of the variability. While JF precipitation in SE Brazil features a weak negative linear trend ($-0.038$ mm/day/year, $\text{p}=0.08$), the trend is positive for the JF South Atlantic 500GPH (0.354 $\text{m}^\prime /\text{year}, \text{p}=0.04$), additionally hinting at a dynamic relation between the two variables. Comparing Figs. 1c and 3a indicates that especially during years when precipitation exceeds one standard deviation below (above) the mean, the 500GPH anomaly is positive (negative). Similarly, when 500GPH exceeds one standard deviation above (below) the mean, precipitation is negative (positive). Further, the six years with 500GPH above one standard deviation (Fig. 3c) exhibit precipitation anomalies in the South Atlantic that are similar to the 2014 case. The same applies to composite 500GPH anomalies in the South Atlantic for the six years with precipitation below one standard deviation (Fig. 3b).

The 2014 South Atlantic blocking event was remarkably persistent as its characteristics are visible in the two-monthly averages. However, since synoptic-scale variability plays an important role in blocking episodes, the correlation analysis is repeated using daily data. The suggested relation between JF South Atlantic 500GPH and SE Brazil OLR (as a proxy for precipitation) holds since a statistically significant Pearson correlation coefficient of 0.39 (p $<0.0001$) for the time period 1982–2019 is found (see Figure S2 in the Supplementary Material). The analysis with daily data additionally indicates that higher South Atlantic 500GPHs tend to coincide with anomalously warm South Atlantic SSTs ($\text{r}=0.52$, p $<0.0001$). Figure 3d illustrates the daily evolution of 10-day running mean SST and 500GPH anomalies averaged over the study area in the South Atlantic from October 2013 to March 2014. From October until mid-December 2013, the South Atlantic 500GPH anomalies fluctuated between positive and negative values. During the same period, South Atlantic SST anomalies were below zero except for the second half of October. In early December 2013, 500GPH anomalies started to increase, which, in agreement with findings by Rodrigues et al. (2019), implies that the blocking anomaly started to develop before the strong SST anomalies. By mid-December, the 500GPH anomalies had turned positive. Starting in mid-January 2014, the 500GPH anomalies rapidly increased close to remarkable $60\,\text{m}^\prime$ above the mean. The 500GPH anomalies then remained highly positive until mid-February, when they dropped to values close to $0\,\text{m}^\prime$, increasing again to about $20\,\text{m}^\prime$ in the second half of February. The variability of the SST anomalies in December, January and February seems to strongly respond to the 500GPH anomalies by roughly following a similar but time-lagged pattern. Furthermore, the present study finds that South Atlantic SST anomalies and SE Brazil OLR anomalies are positively correlated ($\text{r}=0.40$, p $<0.0001$, see Figure S2 in the Supplementary Material), which, in principle, supports findings by Zou et al. (2018), who suggest a positive feedback of high South Atlantic SSTs on negative SE Brazil precipitation. It is, however, difficult to infer causality from this statistical analysis. Therefore, the suggested relations are further investigated using numerical experiments.

### Model results

Having assessed the connection between the South Atlantic 500GPH and SE Brazil precipitation in observations, idealized numerical experiments with an atmospheric general circulation model are used to further investigate whether the 2013/2014 SST anomalies in the tropical and South Pacific, the Indian Ocean and also locally in the South Atlantic have contributed to the anomalous circulation leading to the 2014 SE Brazil drought. Accordingly, the 500GPH responses for the different SST forcings described in Sect. 2.4 are analyzed using the Isca Model (Vallis et al. 2018) to further investigate remote influences on the 2014 South Atlantic anticyclone.

**Fig. 4:**
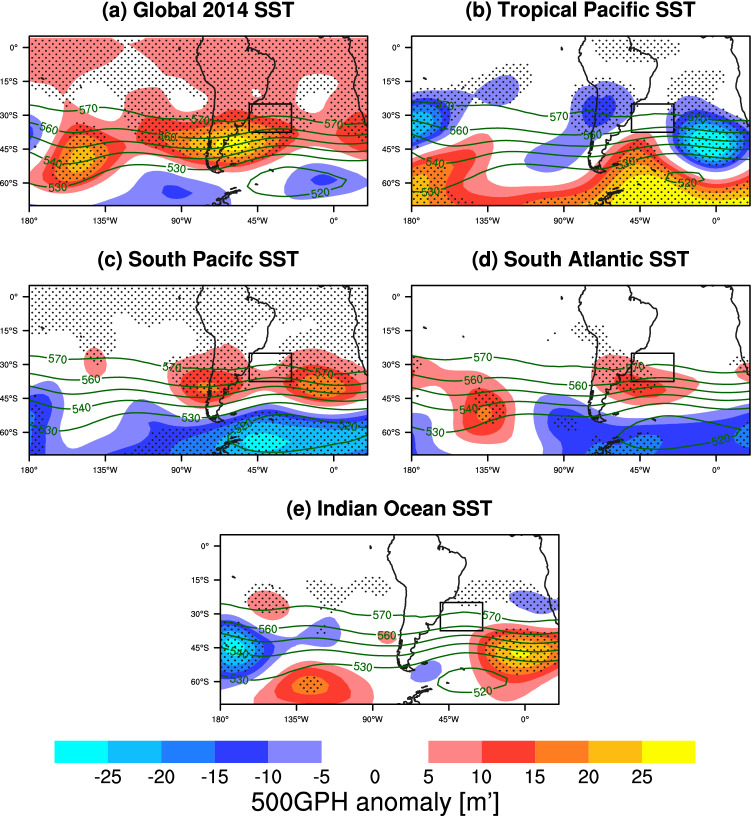
JF ensemble mean 500GPH model response to the **a** globally prescribed 2013–2014 SST seasonal cycle, **b** only the tropical Pacific, **c** the South Pacific, **d** the South Atlantic, and **e** the Indian Ocean. See Fig. 2d for the SST forcing areas. Anomalies are computed with respect to the climatological run. Contours denote the total field (in dam) for each of the SST forced simulations. Significant geopotential height anomalies with respect to the climatological run at the 5% level are dotted

The JF 500GPH ensemble mean response to the different regional SST forcings (see boxes in Fig. 2d) is shown in Fig. 4 and can be compared to reanalysis data (Fig. 2c). Note that the ensemble mean response does not exclusively represent the events that produced elevated 500GPHs off the coast of South America, but it encompasses the full variability of the model response. Forcing the model with the global SST anomalies observed during the austral spring and summer season 2013/2014 is shown to result in a shift of the distribution, indicating a higher likelihood of positive JF 500GPH anomalies over the South Atlantic (Fig. 4a). Forcing 2013/2014 SST anomalies in the tropical Pacific basin only (Fig. 4b) does not yield significant JF 500GPH anomalies in the South Atlantic box. However, when imposing the extratropical South Pacific 2013/2014 SST anomalies (Fig. 4c) to force the model, the distribution is again shifted towards positive 500GPH anomalies over the South Atlantic Brazilian coast. Interestingly, tropical and extratropical Pacific SST anomalies have opposite effects on the South Atlantic atmospheric circulation, with meridional dipoles of opposite sign. When only the SST anomalies in the South Atlantic are forced (Fig. 4d), a meridional dipole over the South Atlantic similar to the one induced by the South Pacific SST forcing is observed. However, the South Atlantic forcing circulation response is composed of one positive 500GPH anomaly, while two positive 500GPH centers are found along $40^\circ \text{S}$ in case of the South Pacific experiment. In contrast, no significant South Atlantic 500GPH anomalies can be observed as a response to an SST forcing in the Indian Ocean (Fig. 4e).

**Fig. 5:**
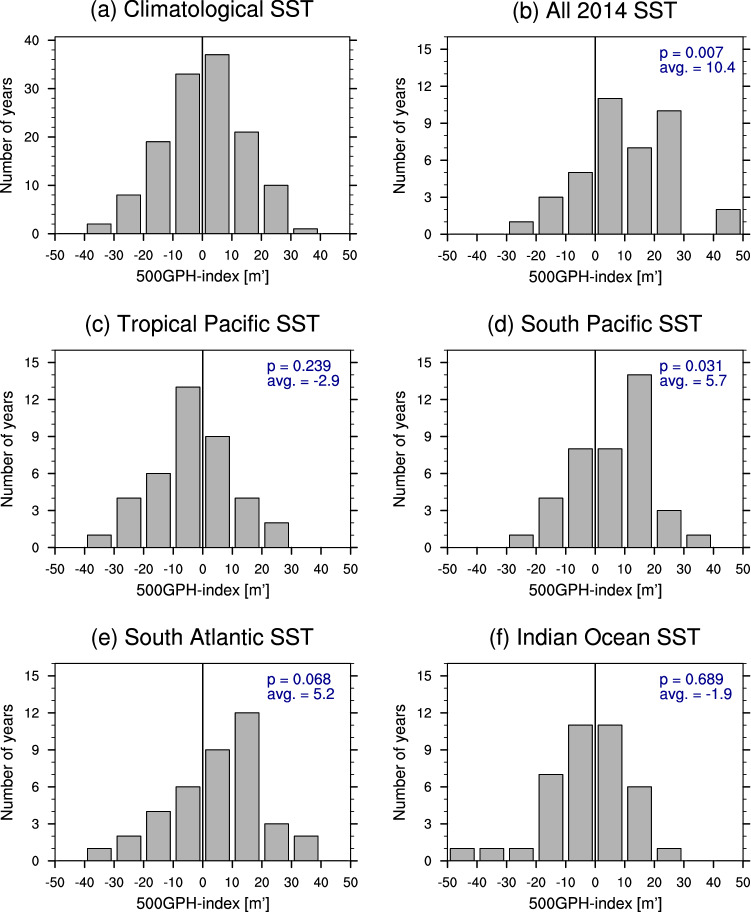
Histograms of JF mean 500GPH anomalies averaged over the South Atlantic anticyclone region [black box in Fig. 4] for **a** the climatological SST run and **b**–**f** the sensitivity experiments with different 2013/2014 SST forcings. Together with the mean values, the p-values obtained from a Kolmogorov–Smirnov test comparing each experiment’s distribution with the climatology (**a**) is shown in the upper right corner of each plot

The area averaged 500GPH index, computed as described in Sect. 2.3, is used to further investigate the 2013/2014 SST anomaly contribution from the different regions to the development of the South Atlantic 500GPH. The area is shown in Fig. 4, and is the same as the dashed box in Fig. 2c. Similar to the analysis with reanalysis data, the standard deviation for the area averaged 500GPH index of the climatological run, i.e. $14\,\text{m}^\prime$, is used to evaluate the occurrence frequency of increased South Atlantic 500GPH for each sensitivity experiment. Figure 5a–f exhibit the distribution of the 500GPH index for JF means for each of the model simulations. In general, these distributions show that internal variability in this region is important, as a wide range of anomalies occur even when the SST forcing is fixed to climatology (Fig. 5a), with 11.5% of ensemble members featuring strongly increased 500GPH in the study area. Figure 5b confirms the relevant role of global SST anomalies in increasing the likelihood of positive 500GPH anomalies over the South Atlantic as the distribution is skewed towards positive values. According to a Kolmogorov–Smirnov test, the distribution obtained is statistically significantly different above the 99% level compared to the climatological SST experiment. The contribution of global SSTs is also reflected in the corresponding occurrence frequency of 35.9%, which is the highest among the experiments. With 25.6 and 23.1% of ensemble members exceeding the threshold for strongly increased 500GPH when forcing the model with the South Pacific (Fig. 5d) or South Atlantic (Fig. 5e) SST anomalies, respectively, the distributions peak at positive anomalies. Hence, this analysis supports their positive contribution to the blocking event, with the respective 500GPH distributions being statistically different from the climatology at the 95 and 90% confidence level. On the other hand, the distribution confirms an insignificant role or even negative contribution from the tropical Pacific (Fig. 5c) and Indian Ocean (Fig. 5f), as their corresponding distributions are skewed towards negative South Atlantic 500GPHs. Both experiments exhibit a decrease in the occurrence frequency of strongly elevated South Atlantic 500GPH compared to the other sensitivity experiments with 15.4% for the Indian Ocean and 10.3% for the tropical Pacific simulation, which is less than for the climatological simulation in the latter case.

**Fig. 6:**
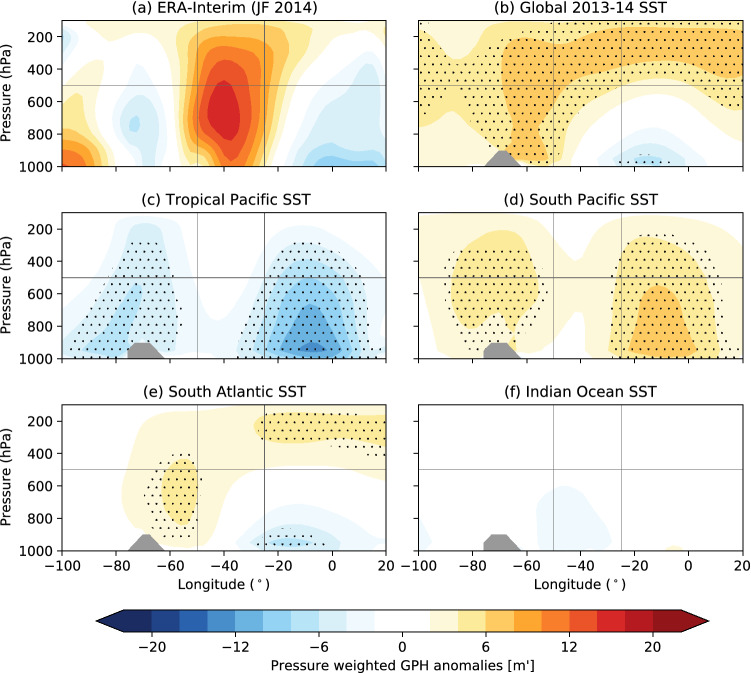
JF mean longitude-pressure cross-sections of pressure weighted geopotential height for **a** ERA-Interim reanalysis data and **b**–**f** the sensitivity experiments with different 2013/2014 SST forcings for the ensemble mean. The vertical grey lines reflect the longitudinal borders of the study area and the horizontal one the 500 hPa level. Significant geopotential height anomalies with respect to the climatological run at the 5% level are dotted. GPH anomalies are multiplied by the p/1000 factor, where p is the pressure level in hPa

The vertical structure of exceptionally strong 500GPH anomalies over the South Atlantic is evaluated in the following. Figure 6 shows the longitude-pressure cross sections of the JF pressure weighted geopotential height anomalies, i.e. anomalies are multiplied by *p*/1000, where p is the pressure level in hPa. These are shown for ERA-Interim reanalysis (Fig. 6a) and for the sensitivity experiments (Fig. 6b–f). Note that these sensitivity experiments are highly idealized and not meant to exactly reproduce the observed event, but instead they are used to evaluate the likelihood of increased 500GPH over the South Atlantic. The vertical structures of the anticyclonic anomaly exhibit some variety between the different model experiments. In reanalysis data (Fig. 6a), the barotropic nature of the blocking event is clearly visible as South Atlantic positive 500GPH anomalies extend across the full tropospheric column with a relative maximum at about 650 hPa. On average, the model produces negative (positive) 500GPH anomalies in the lower (upper) troposphere over the South Atlantic in the global 2013/2014 SST (Fig. 6b) and the South Atlantic (Fig. 6e) SST experiment, which might be related to increased evaporation at lower levels and condensation at upper levels due to increased convection triggered by the local warm SST anomalies. Tirabassi et al. (2015), for example, highlight the importance of a regional coupling between the ocean and the atmosphere over the South Atlantic and the high variability in the direction of the coupling between the ocean and the atmosphere. In the model, the representation of processes is limited to the influence of SSTs onto the atmosphere. Thus, the feedback of the atmosphere onto the SSTs is not represented and might be one of the factors that explain why the model cannot fully reproduce the barotropic structure of the 2014 blocking over the study area in the South Atlantic. Nevertheless, using a more complex atmospheric-ocean model does not guarantee a better understanding of the processes and the simulations are useful in assessing the role of the 2013/2014 SSTs for the circulation over the South Atlantic. In fact, both experiments exhibit a barotropic anticyclone to the west of the study area around $60^\circ \text{W}$. The vertical structure of the 2014 barotropic anticyclone over the study area is, however, better represented in the South Atlantic experiment. Thus, locally prescribed high SSTs may help establish the blocking pattern over the South Atlantic. The ensemble mean response to the South Pacific SST forcing (Fig. 6d) features two barotropic 500GPH anomalies to the west and east of the study area, respectively, with the latter overlapping its eastern part. Neither the ensemble mean response of the tropical Pacific experiment (Fig. 6c) nor of the Indian Ocean experiment exhibit positive 500GPH across the vertical column of the study area. While the longitude-pressure cross sections of the JF pressure weighted geopotential height anomalies, for the composite of ensemble members which feature a South Atlantic 500GPH stronger than 1 standard deviation of the climatological model run (i.e. $14\,\text{m}^\prime$) (see Figure S3 in the Supplementary Material) instead of the ensemble mean reflect that each sensitivity experiment includes ensemble members that feature significant elevated 500GPH in the study area, the occurrence frequency, and thus, the likelihood of occurrence differs for each model run. Nevertheless, the analysis underlines that the composite of strong 500GPH anomalies of the South Atlantic SST simulation best represents the vertical structure of the observed event in 2014 including the barotropic structure and the mid-tropospheric maximum.

**Fig. 7:**
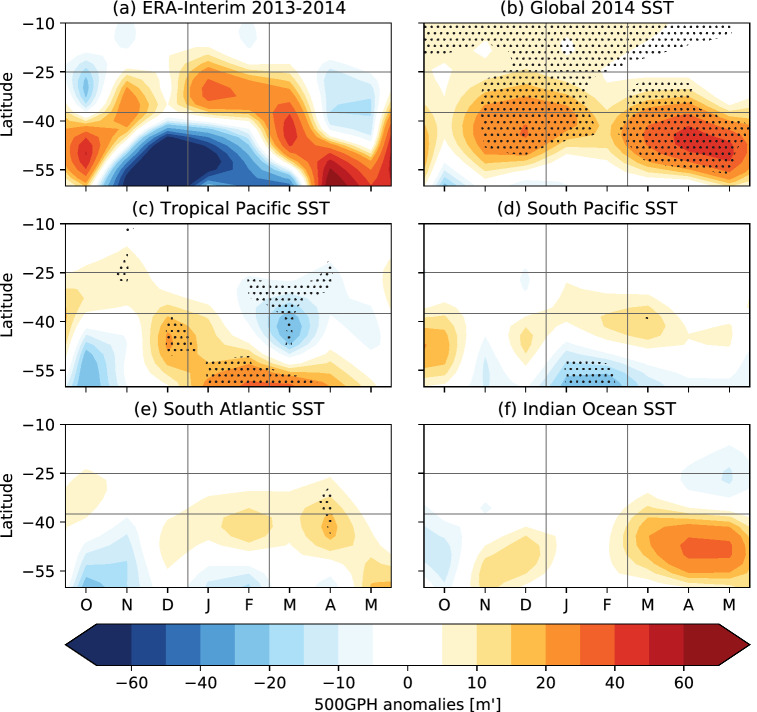
Time-latitude evolution of the monthly mean 500GPH anomalies zonally averaged over the study area [$50{-}25^\circ \text{W}$] for **a** ERA-Interim reanalysis data and **b**–**f** the sensitivity experiments with different 2013/2014 SST forcings for the ensemble mean. The vertical black lines reflect the JF time period and the horizontal ones the latitudinal borders of the study area. Significant 500GPH anomalies with respect to the climatological run at the 5% level are dotted

The ensemble mean time-latitude evolution of monthly mean 500GPH anomalies zonally averaged over the study area is shown in Fig. 7b–f for the different sensitivity experiments. ERA-Interim reanalysis data for JF 2014 (Fig. 7a) shows that the anticyclonic anomaly sets up in the study area in late December, reached its maximum in mid-January and kept its latitudinal position until the end of February before it began to move poleward. In the global SST experiment (Fig. 7b) an anticyclonic anomaly develops in early November which starts to migrate southward in early February. Among the sensitivity experiments, the experiment with a global 2013/2014 SST forcing (Fig. 7b) shows the strongest response over the South Atlantic at the beginning of January. Note that the 500GPH anomalies seem to have a poleward shifted offset when compared to reanalysis, which is also observed in the South Pacific (Fig. 7d) and South Atlantic (Fig. 7e) experiment. In late December, positive South Atlantic 500GPHs develop in the South Pacific and South Atlantic experiment which feature a meridional structure with negative anomalies poleward of positive 500GPH anomalies over the study. Furthermore, the poleward migration of the positive anomaly that is observed in reanalysis data is approximately replicated. The ensemble mean time latitude evolution for the tropical Pacific (Fig. 7c) and Indian Ocean (Fig. 7f) experiment do not show positive anomalies developing in the study area during JF.

In conclusion, our numerical simulations suggest that the tropical Pacific SSTs alone are unlikely to have contributed to the South Atlantic blocking episode in JF as the occurrence frequency of strong South Atlantic 500GPH events is very low in our experiment. Furthermore, while the ensemble members that do produce elevated 500GPH in the study area exhibit a time evolution of the event that resembles the 2014 case in reanalysis data (see Figure S4 in the Supplementary Material), the likelihood for a contribution from the Indian Ocean alone is small as well. The 2013/2014 South Pacific SST forcing shows the largest 500GPH response in JF in the ensemble mean (Fig. 5d) coinciding with the observed event. Interestingly, the vertical structure of JF 500GPH events in the South Atlantic model run might indicate an involvement of local SSTs in setting the vertical structure of the blocking. Thus, these model results suggest that while neither the Indian Ocean nor the tropical Pacific SSTs alone are likely to have contributed to the onset of the blocking event during the 2014 SE Brazilian drought, the South Pacific SST forcing might have been strongly involved in generating enhanced 500GPH during the peak of the event. Besides, South Atlantic SSTs might have contributed to the vertical setup of the event.

## Summary and discussion

The lack of rainfall in January–February 2014 in SE Brazil severely impacted the state’s agriculture, resulting in major economic losses (e.g. Benfield 2015). The precipitation rate in the study area exhibited the lowest recorded value in 41 years. The analysis of the JF 2014 SLP and 500GPH reveals an intensified and displaced subtropical high with a barotropic structure in the lower troposphere. This anticyclonic anomaly in the South Atlantic altered the circulation and inhibited precipitation in SE Brazil. Consistent with Seth et al. (2015), considerable warm SST anomalies appear alongside this South Atlantic blocking high. Additionally, the South Atlantic 500GPH and SE Brazil precipitation are found to be significantly negatively correlated, which confirms findings by Rodrigues and Woollings (2017). In particular, extremely dry events in the study area coincide with strongly enhanced 500GPH anomalies, justifying the use of 500GPH as an indicator for precipitation in SE Brazil, whose sensitivity to SST forcing in various regions is analyzed with idealized numerical experiments in the present study. The model simulations reveal a large variability among the ensemble members. Hence, internal atmospheric variability is very important for determining the atmospheric pattern in this region. The probability of higher South Atlantic 500GPH is most strongly increased when imposing the global 2013/2014 SST anomaly evolution to force the model. SST anomalies (Fig. 2d) coincide with convective anomalies detected in the western tropical Pacific (Coelho et al. 2016), potentially favoring the development of barotropic Rossby waves propagating toward the South Atlantic. However, the model response indicates an insignificant role of tropical Pacific forcing in generating enhanced 500GPH anomalies in the South Atlantic. In addition to a signal arising from the western tropical Pacific, convection over the Indian Ocean associated with the MJO has been suggested to drive a Rossby wave train featuring a phase similar to the 2014 case, unfavorable for the development of SACZ episodes (Rodrigues and Woollings 2017). However, forcing the model with 2013/2014 Indian Ocean SST anomalies alone is not found to significantly increase the probability of enhanced South Atlantic 500GPH anomalies. Since the convective response associated with the MJO is generally uncoupled from the underlying SSTs, we cannot rule out the potential role of the MJO in the 2014 Brazil drought. The RMM index indicates that the MJO was briefly in a phase that, according to Rodrigues and Woollings (2017), is conducive to the generation of South Atlantic blocking in the early austral summer season and thus could have been involved in initiating a blocking event. However, the MJO was not active during the onset and the two weeks preceding the 2014 blocking event. Therefore, another factor must have contributed to the persistence of the blocking anomalies in the South Atlantic. Our experiments show that the South Pacific has likely acted as an essential contributor to the increased 500GPH in the study area. This is consistent with the findings of Coelho et al. (2016) who show that the South Pacific contributed to establishing the extratropical component of the wave train to the South American region. The Indian Ocean and the tropical Pacific might have been indirectly involved as signals from both regions might have modified the South Pacific SSTs with which the model is forced. Thus, the results may reveal their potential synergy. Our results show that in addition, warm South Atlantic SSTs tend to coincide with increased OLR over SE Brazil. This is consistent with findings by Barreiro et al. (2019), who demonstrate that warm South Atlantic SSTs cause a southward shift of the SACZ by promoting low-level convergence over the South Atlantic and thus locally modify the regional atmospheric circulation by air–sea coupling. Furthermore, the South Atlantic SST forcing causes a positive increase in the JF likelihood of high 500GPH anomalies whose vertical structure closely resembles the 2014 case. Thus, local SST anomalies generated by the South Atlantic atmospheric circulation in the first place (Rodrigues et al. 2019) might have been involved in arranging the vertical structure of the blocking event. In conclusion, while the global 2013/2014 SSTs are likely to have contributed to the South Atlantic blocking that caused the unusually negative precipitation anomalies in SE Brazil, neither tropical Pacific nor Indian Ocean SSTs and the corresponding teleconnections alone are found to have been able to account for the observed circulation anomaly. Instead, SST anomalies in the South Pacific, as well as local feedbacks with South Atlantic SSTs are found to have likely contributed to the blocking event. The present study reconciles the previous literature on the SE Brazil drought in the sense that the MJO could have been the initial trigger of the Rossby wave train (Rodrigues and Woollings 2017; Rodrigues et al. 2019; Barreiro et al. 2019) that was reinforced over the South Pacific to reach South America (Coelho et al. 2016), while local South Atlantic SSTs (Zou et al. 2018; Barreiro et al. 2019) might have helped to prolong the drought during the summer season. The responses to the imposed SST forcings in the model also indicate a high internal atmospheric variability. Atmosphere–ocean feedbacks, which cannot be represented in the model, may have further influenced the extreme conditions observed in 2014. Thus, the present study emphasizes the complex nature of the observed drought in SE Brazil and suggests a combination of several influences and their potential non-linear interactions together with internal atmospheric variability as an explanation for the occurrence of such a significant and persistent event.

## Electronic supplementary material

Below is the link to the electronic supplementary material.

Supplementary material 1 (pdf 703 KB)
