# Supplementary material for: Revisiting remote drivers of the 2014 drought in South-Eastern Brazil
Source: Clim Dyn. 2020 Sep 3;55(11):3197–211. doi: 10.1007/s00382-020-05442-9 (PMC7572352; doi:10.1007/s00382-020-05442-9)
Supplement: Supplementary file 1 — Supplementary material 1 (pdf 703 KB) [file 382_2020_5442_MOESM1_ESM.pdf]

Supplementary Material

Revisiting remote drivers of the 2014 drought in South-Eastern Brazil

Climate Dynamics

Kathrin Finke · Bernat Jiménez-Esteve · Andréa S. Taschetto · Caroline C. Ummenhofer · Karl Bumke · Daniela I. V. Domeisen

Kathrin Finke
Department of Meteorology, Stockholm University, Stockholm, Sweden

Bernat Jiménez-Esteve
Institute for Atmospheric and Climate Science, ETH Zurich, Zurich, Switzerland

Andréa S. Taschetto
Climate Change Research Centre, University of New South Wales, Sydney, Australia
ARC Centre of Excellence for Climate Extremes, University of New South Wales, Sydney, Australia

Caroline C. Ummenhofer
Department of Physical Oceanography, Woods Hole Oceanographic Institution, Woods Hole, USA
ARC Centre of Excellence for Climate Extremes, University of New South Wales, Sydney, Australia

Karl Bumke
Marine Meteorology Department, GEOMAR Helmholtz Centre for Ocean Research Kiel, Kiel, Germany

Daniela I.V. Domeisen
Institute for Atmospheric and Climate Science, ETH Zurich, Zurich, Switzerland
E-mail: daniela.domeisen@env.ethz.ch

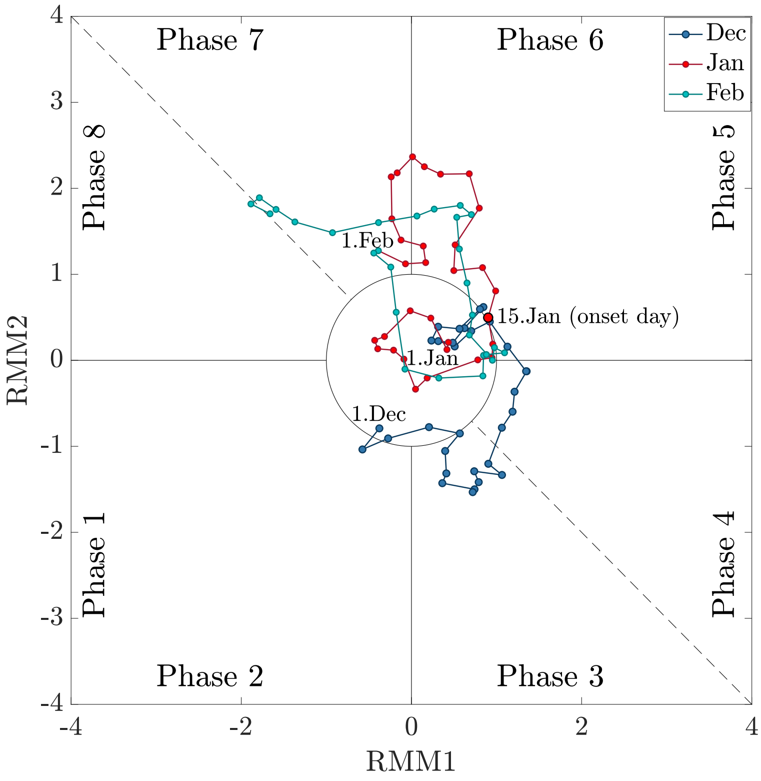

Fig. S1 MJO phase evolution based on RMM index for December 2013 (blue), January 2014 (red) and February (2014). The onset day of the South Atlantic blocking event as proposed by Rodrigues et al. (2017), i.e. Jan 15th, 2014, is marked in the Figure.

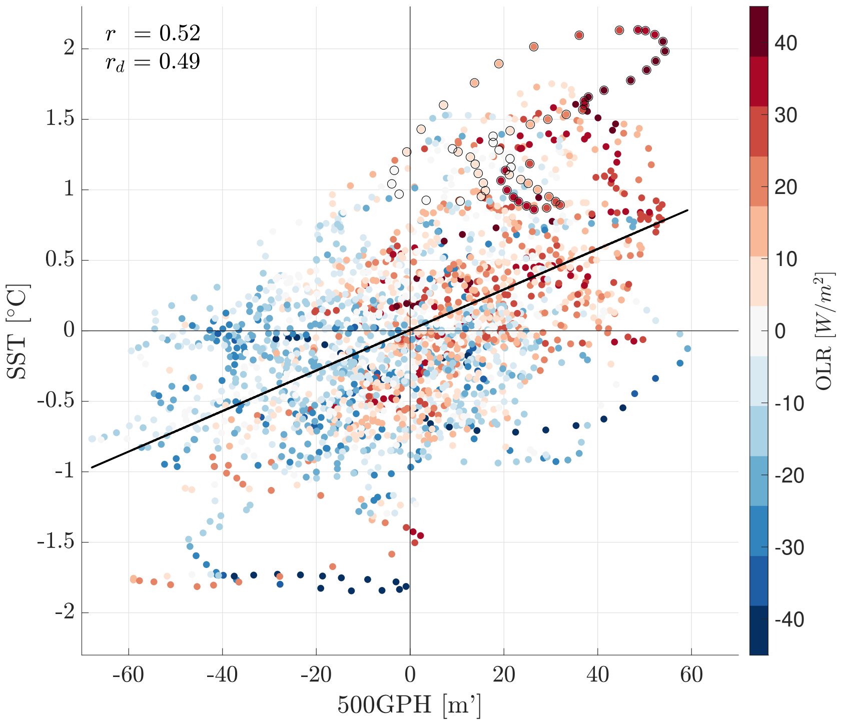

Fig. S2 Relation between JF South Atlantic 500GPH, South Atlantic SST and SE Brazil OLR anomalies (color). A 10-day running mean was applied to the data before averaging over the respective region. Black circled dots represent values for JF 2014. r (r_d) denotes the Pearson correlation coefficient between the (detrended) JF 500GPH and SST time series. All data cover the time period from 1982 to 2019.

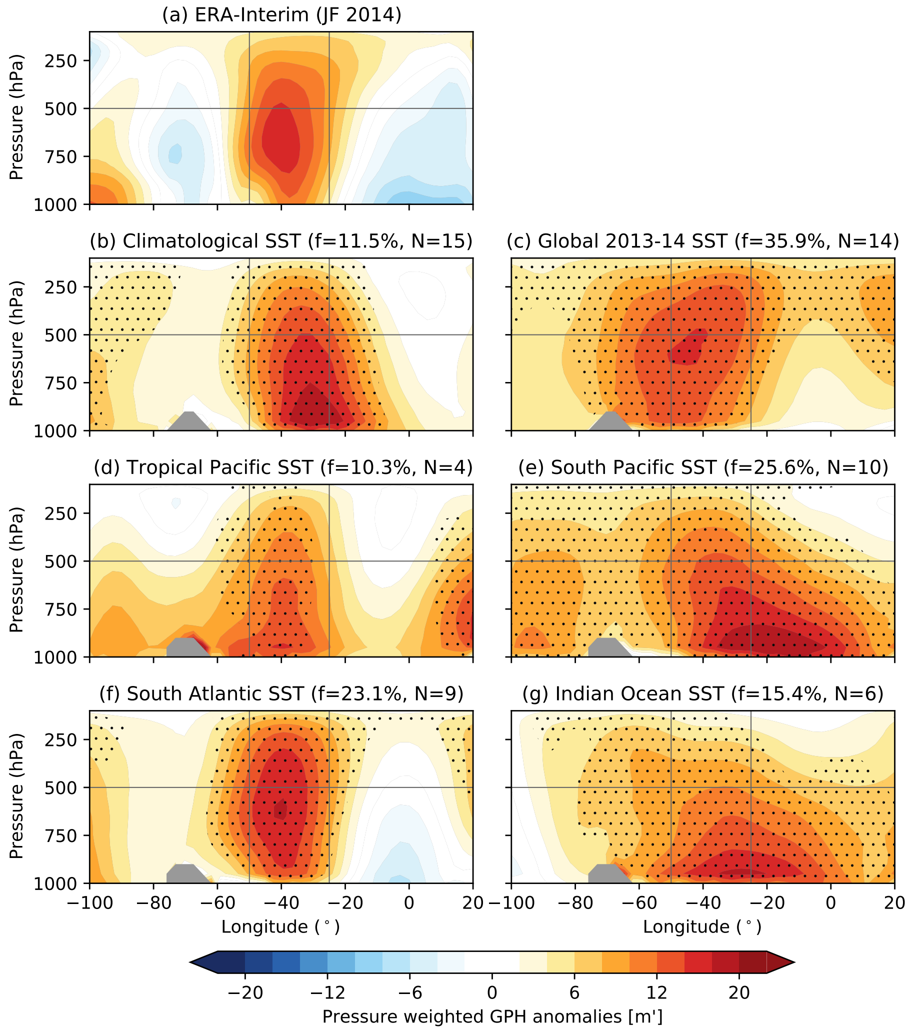

Fig. S3 JF mean longitude-pressure cross-sections of pressure weighted geopotential height for a) ERA-Interim reanalysis data, b) the climatological SST run and (c-g) the sensitivity experiments with different 2013/2014 SST forcings composited for a JF South Atlantic 500GPH index exceeding 1 standard deviation of the climatological model run ($14m'$). The number of ensemble members N fulfilling this criterion and corresponding occurrence frequency are indicated in each title. The vertical grey lines reflect the longitudinal borders of the study area and the horizontal one the 500hPa level. Significant geopotential height anomalies with respect to the climatological run at the 5% level are dotted.

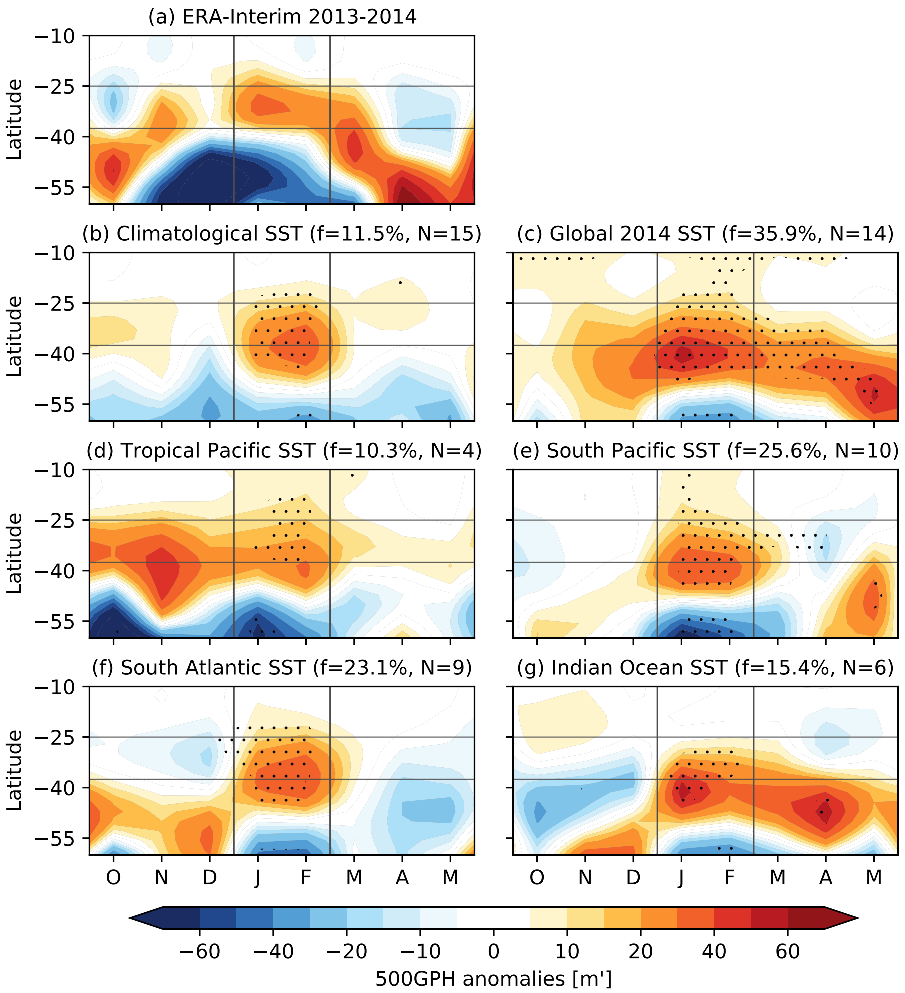

Fig. S4 Time-latitude evolution of the monthly mean 500GPH anomalies zonally averaged over the study area [50-25°W] for a) ERA-Interim reanalysis data b) the climatological SST run and (c-g) the sensitivity experiments with different 2013/2014 SST forcings composited for a JF South Atlantic 500GPH exceeding 1 standard deviation of the climatological model run ($14m'$). The number of ensemble members N fulfilling this criterion and corresponding occurrence frequency are indicated in each title. The vertical black lines reflect the JF time period and the horizontal ones the latitudinal borders of the study area. Significant geopotential height anomalies with respect to the climatological run at the 5% level are dotted.
